# Hidden Hypergraphs, Error-Correcting Codes, and Critical Learning in Hopfield Networks

**DOI:** 10.3390/e23111494

**Published:** 2021-11-11

**Authors:** Christopher Hillar, Tenzin Chan, Rachel Taubman, David Rolnick

**Affiliations:** 1 Awecom, Inc., San Francisco, CA 94103, USA; taubmanrachel@gmail.com; 2 Singapore University of Technology and Design, Singapore 487372, Singapore; tenzin_chan@mymail.sutd.edu.sg; 3 School of Computer Science, McGill University, Montreal, QC H3A 0G4, Canada; drolnick@cs.mcgill.ca

**Keywords:** Hopfield networks, clustering, error-correcting codes, exponential memory, hidden graph, neuroscience

## Abstract

In 1943, McCulloch and Pitts introduced a discrete recurrent neural network as a model for computation in brains. The work inspired breakthroughs such as the first computer design and the theory of finite automata. We focus on learning in Hopfield networks, a special case with symmetric weights and fixed-point attractor dynamics. Specifically, we explore minimum energy flow (MEF) as a scalable convex objective for determining network parameters. We catalog various properties of MEF, such as biological plausibility, and then compare to classical approaches in the theory of learning. Trained Hopfield networks can perform unsupervised clustering and define novel error-correcting coding schemes. They also efficiently find hidden structures (cliques) in graph theory. We extend this known connection from graphs to hypergraphs and discover *n*-node networks with robust storage of $2^{\Omega (n^{1-\epsilon })}$ memories for any ϵ>0. In the case of graphs, we also determine a critical ratio of training samples at which networks generalize completely.

## 1. Introduction

In their seminal work, McCulloch and Pitts [1] developed a theory of discrete recurrent neural networks (DRNNs) that simultaneously contained a model for spike trains (sequences of action potentials in neural activity), a computational theory of mind [2], and the start of circuit design for programmable electronic computers [3]. Many variations of these concepts have since guided research in artificial intelligence and neuroscience. We shall focus here on the problem of learning in the special case of Hopfield networks [4], which are McCulloch–Pitts networks with symmetric weights having dynamics on states that always result in fixed-point attractors. Such patterns that persist under the dynamics [5] are considered to be the memories of the network.

Much attention in machine learning research in the last decade has been devoted to supervised multi-layer feedforward networks [6]. More recently, though, it has been found that shallow models [7], and in particular, classical ones such as the Hopfield network can help simplify architectures in deep learning. For instance, the work of [8] links attractor networks to deep learning and transformers [9,10]. These findings also bring the field closer to biology, where recurrence seems to be a fundamental property of neuronal circuits [11,12]. Additionally, neuroscience has benefited from the application of single-layer maximum entropy models [13]. In particular, it has been shown that retinal spiking output [14,15] is well-described by a second-order Lenz–Ising distribution [16], which is the underlying maximum entropy model for Hopfield networks.

**Figure 1:**
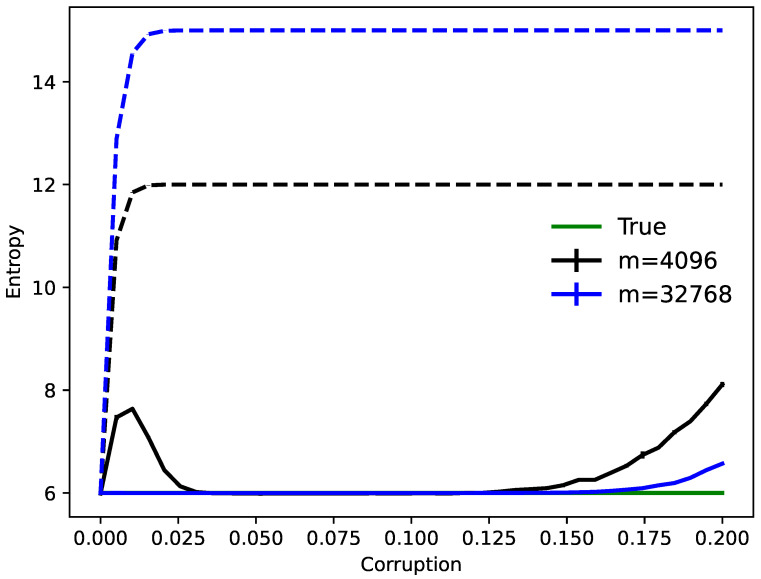
How many clusters? The entropy of corrupted binary distributions are estimated by learning DRNNs. Over several trials, $2^{6}=64$ binary vectors in dimension n=256 are randomly chosen as hidden cluster centers. Independent samples of sizes m=4096 and m= 32,768 taken from these originals are corrupted by independently changing bits with increasing probability. Using MEF to obtain a Hopfield network, dynamics converges data points to fixed points, and the Shannon entropy of these is calculated (SD errors) versus that of corrupted samples (dashed lines).

**Figure 2:**
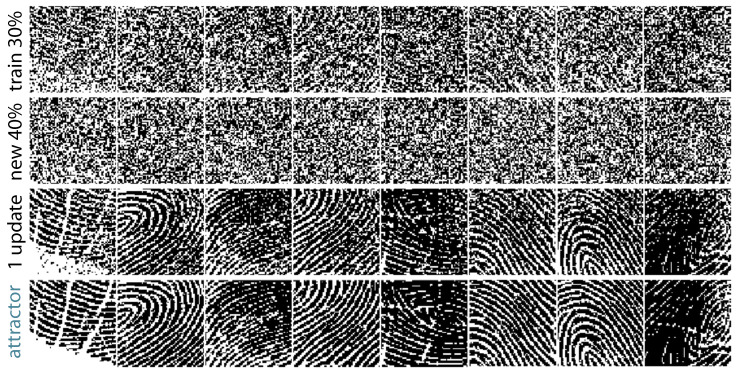
Hidden fingerprints. Unsupervised clustering of corrupted versions of eighty 4096-bit (64×64) human fingerprints [21]. From top row to bottom (each column represents a different fingerprint): one sample of a 30% corrupted fingerprint shown during learning, novel 40% corrupted fingerprint shown to network after training, result of one iteration of dynamics initialized at a novel pattern, and converged fixed-point attractor bit-for-bit identical to the original fingerprint.

More generally, a fundamental challenge in data science is to uncover and model the latent causes generating a set of measurements. We show how to learn Hopfield networks that can be used to solve this problem and outline several experimental and theoretical findings. Our main tool is a convex learning objective called minimum energy flow (MEF), defined in Section 3 (see Definition 1), which has many useful properties. For instance, networks trained with MEF can perform unsupervised clustering and denoising (Figure 1 and Figure 2). Moreover, MEF learning is biologically plausible (Section 3.6).

Another classical problem is to find networks that store a large number of memories, all with large basins of attraction. Such networks determine practical (nonlinear) error-correcting coding schemes. Several solutions to this problem have recently appeared demonstrating robust exponential capacity in Hopfield networks [17,18,19,20]. We extend the results of [19] from the graph case to that of hypergraphs (Theorem 2), which allows us to construct *n*-node networks with robust storage of $2^{\Omega (n^{1-\epsilon })}$ memories for any ϵ>0.

It was also observed in [19] (Figure 2) that there is a critical ratio of training samples to total number of patterns at which complete storage of all patterns occurs. Here, we investigate this phenomenon deeper and provide evidence that the critical ratio decays exponentially with the number of vertices (Conjecture 1).

The paper is organized as follows. In Section 2, we give an outline of some applications that are touched upon by this work. In Section 3, we present the requisite background for Hopfield networks and minimum energy flow learning, including a new inequality relating MEF to probability density estimation (Theorem 1). Our main results appear in Section 4, which include an application to experimental neuroscience as well as precise statements of main theoretical and computational findings. Next, in Section 5, we give detailed proofs of our mathematical results. Finally, we close with a discussion in Section 6 followed by a short conclusion in Section 7.

## 2. Applications

The main motivation for this work was to extend the theory of learning and memory capacity in Hopfield DRNNs, which at a high level can be viewed as denoising autoencoders for binary variables. However, the setup is sufficiently general to apply to clustering, signal modeling, error-correcting codes, graph theory, and learning theory. We briefly outline several of these applications of Hopfield networks.

In a typical example, an underlying true distribution is sampled then corrupted with noise, and the goal is to learn network parameters (weights, thresholds) uncovering the original distribution and sources (Figure 1 and Figure 2). The recurrent dynamics can be used to autoencode or label any new data point with its fixed-point attractor (Figure 2), and these labels are interpreted as the network’s best guesses for latent structure in the samples.

### 2.1. Unsupervised Clustering

A classical problem in data science is to determine the number of true sources or clusters that generate a specific set of samples [22], ideally with as few assumptions as possible. For instance, in the specific problem of image category labeling, unsupervised deep learning approaches have been found to be powerful [23]. Many other attacks on the problem are possible, including hidden Markov models with Bayesian expectation–maximization [24,25] and dimensionality reduction with PCA [26], among others [27]. We investigate minimizing the energy flow objective function (Definition 1) over unlabeled data sets to obtain Hopfield networks that cluster them.

As a simple example, consider a source distribution supported on several binary vectors (the hidden clusters) in dimension *n* and assume access to it only through *m* noisy samples. After training, we may estimate the Shannon entropy [28] of the original distribution by calculating the entropy over the fixed points determined by dynamics initialized at the data. The results are plotted in Figure 1 for a particular setup. Note that when both the sample size and corruption level are small, this entropy estimate is inaccurate since noisy original clusters are stored as distinct memories. However, with a sufficient number of samples *m*, the estimate matches the underlying truth.

The general success of entropy estimation with this method is intimately connected to whether the underlying causes in the data are being correctly or approximately autoencoded by the network. One way to illustrate this observation is by generating noisy samples as before but with the hidden sources arising from natural image data.

In Figure 2, we summarize the results of such an experiment. A set of binarized human fingerprints was corrupted with significant noise (top row in Figure 2), and a Hopfield network was trained with MEF on these data. Having never seen original fingerprints and with unlabeled information, the network nonetheless learns each original source as a fixed point with a large basin of attraction. For instance, as shown in Figure 2, dynamics takes 40% corrupted samples (second row) to the exact originals (bottom row).

### 2.2. Natural Signal Modeling

Modeling the structure of signals arising from nature is another classical topic [13]. With the appropriate discretization, a natural signal ensemble can be studied by learning a Hopfield network; for instance, in the pursuit of image compression [29,30], perceptual metrics [31], or rate-distortion analyses [32,33]. These networks and their memories can also be used to understand data from neuroscience experiments [34,35]. In particular, it is possible to uncover reoccurring spatiotemporal activity patterns in spontaneous neural activity. We explain this finding in Section 4.1. The software package HDNET [36] was used to perform analyses, and it is a general tool for neuroscience that includes neural modeling with MEF and Hopfield networks.

### 2.3. Error-Correcting Codes

Each Hopfield network can be thought of as an error-correcting coding scheme about its fixed points. In recent years, there has been much activity [17,18,19,20] finding networks with large memory capacities that also have large basins of attraction around fixed points (so-called robust networks). In particular, it has been shown that there are Hopfield networks with robust exponential capacity (see Section 3.2), and thus can perform practical error-correction. We add to this body of work by generalizing [19] to find new families of error-correcting codes arising from larger attractor sets. See Section 4.2 for more details (specifically, Theorem 2 and Corollary 1).

### 2.4. Computational Graph Theory

A classical approach of [37] is to identify solutions to graph problems, such as finding short paths between vertices, with energy minima in Hopfield networks. An appropriate network could, for instance, give approximate solutions to the Travelling Salesman Problem by converging dynamics initialized at an input graph. More generally, many NP-complete and NP-hard problems can be formulated as finding energy minima in Lenz-Ising models [38], with practical applications leveraging quantum devices [39].

Another basic task in computer science is to efficiently find large cliques in graphs (the NP-complete max clique problem). A simplification of this unsolved challenge is to uncover a single clique that has been hidden with noise, called the hidden clique problem [40]. As a direct consequence of the theory in [19], Hopfield networks can learn to solve this problem by placing each clique as a local energy minimum of the dynamics. Here, we extend this finding to the case of hypergraphs (Theorem 2), thereby providing an efficient DRNN solution to the hidden hyperclique problem.

### 2.5. Theory of Learning

**Figure 3:**
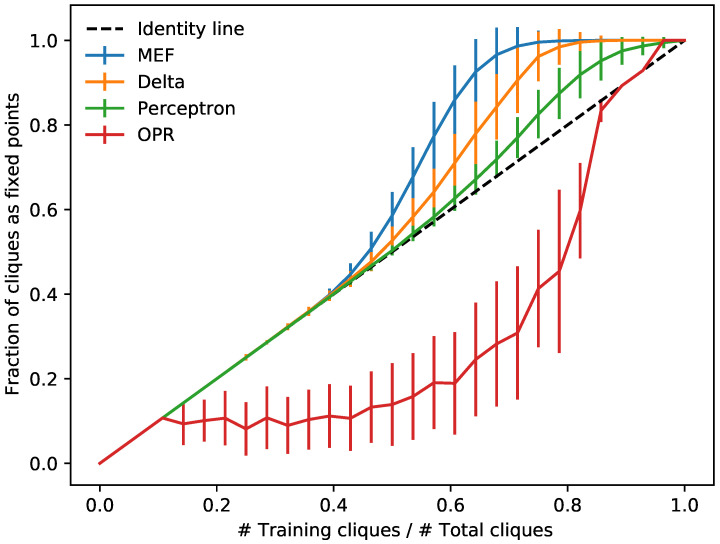
Learning to find hidden cliques. As a function of the ratio of random training samples to total number of patterns to memorize, the fraction of all *k*-cliques in *v*-vertex graphs stored in a Hopfield network on *n* nodes is calculated, trained with the learning rules OPR, perceptron, delta, and MEF (Table 1) using all cliques as a test set (n=28,v=8,k=6; 500 trials, SD errors).

A theory of network computation in brains was formulated in [1], but the problem of learning was largely left open. Several strategies for determining underlying parameters (abstract synaptic weights) in McCulloch–Pitts networks have since appeared such as Hebb [4,41,42], perceptron [43], delta [44,45], and contrastive divergence [46] rules; see Table 1. We explore minimum energy flow in this context and describe several of its useful properties. We also compare it to these classical approaches to learning (Figure 3).

## 3. Background

In this section, we present the abstract model and concepts that will be used throughout the paper, including a theory of learning with minimum energy flow. We also outline the advantages of this approach to training Hopfield networks.

Let $\langle x,y\rangle =x^{⊤}y$ denote the inner product between two column vectors *x* and *y* (we also set $M^{⊤}$ to be the transpose of a vector or matrix *M*). Furthermore, ${∥x∥}_{2}={\langle x,x\rangle }^{1/2}$ and ${∥x∥}_{1}=|x_{1}\left|+\ldots +\right|x_{n}|$ are the $\ell_{2}$ and $\ell_{1}$ norms of *x*, respectively.

### 3.1. Hopfield Networks

Our basic objects are Hopfield networks [4] on *n* binary nodes. Given a real symmetric *weight* matrix $W=W^{⊤}\in R^{n\times n}$ with zero diagonal ($W_{ii}=0$ for all *i*) and a *threshold* vector $\theta \in R^{n}$, an energy function on states $x={\left(x_{1},\ldots ,x_{n}\right)}^{⊤}\in {\left\{0,1\right\}}^{n}$ is defined by:

(1) $$E_{x}=-\frac{1}{2}x^{⊤}Wx+x^{⊤}\theta =-\sum_{i<j}W_{ij}x_{i}x_{j}+\sum_{i=1}^{n}x_{i}\theta_{i}.$$

These weights and thresholds also parameterize a general Lenz–Ising [16] distribution $p={\left(p_{x}\right)}_{x\in {\left\{0,1\right\}}^{n}}$:

(2) $$p_{x}=\frac{e^{-E_{x}}}{Z},\;Z=\sum_{x}e^{-E_{x}}.$$

The Lenz–Ising model is known to have maximum entropy over all distributions with its first- and second-order statistics [47] and often can be determined from very few of its samples [48,49,50].

The pair (W,θ) determines asynchronous deterministic (zero-temperature) linear threshold *dynamics* on states *x* by replacing, in some fixed order, each $x_{i}$ at node *i* with: $x_{i}=1$ if $\sum_{j\neq i}W_{ij}x_{j}>\theta_{i}$; and $x_{i}=0$, otherwise. These dynamics are compatible with the energy function as it does not increase energy ($W_{i}$ is the *i*th column of *W*):

(3) $$\Delta E_{i}=-\Delta x_{i}\left(W_{i}^{⊤}x-\theta_{i}\right).$$

Using (3), one can verify that each initial state $x\in {\left\{0,1\right\}}^{n}$ converges to a fixed-point *attractor* $x^{*}$ in a finite number of such steps through all nodes:

(4) $$x^{*}=H\left(Wx^{*}-\theta \right).$$

Here, *H* is the Heaviside function; that is, H(r)=1 if r>0; and H(r)=0, otherwise.

### 3.2. Robust Capacity

We now formalize the notion of robust memory storage for families of Hopfield networks. The *p-corruption* of *x* is the random pattern $x_{p}$ obtained by replacing each $x_{i}$ by $1-x_{i}$ with probability *p*, independently. The *p*-corruption of a state differs from the original by pn bit flips on average so that for larger *p* it is more difficult to recover the original binary pattern; in particular, $x_{\frac{1}{2}}$ is independent of *x*. Some examples of the *p*-corruption of binary fingerprints for p=0.3 and p=0.4 can be found in Figure 2.

Given a Hopfield network, the fixed-point $x^{*}$ has *(1−ϵ)-tolerance* for a *p*-corruption if the dynamics can recover $x^{*}$ from $x_{p}^{*}$ with a probability of at least 1−ϵ. The *α-robustness* α(X,ϵ) for a set of states *X* is the most *p*-corruption every state (1−ϵ)-tolerates.

Finally, we say that a sequence of Hopfield networks *robustly stores* states $X_{n}$ with robustness index α>0 if the following limit exists and equals α:

(5) $$\lim_{\epsilon \to 0^{+}}\lim_{n\to \infty }\alpha \left(X_{n},\epsilon \right)=\alpha .$$

Intuitively, if α is the robustness index, then the chance that dynamics do not recover a *p*-corrupted memory, p<α, can be made as small as desired by devoting more neurons.

### 3.3. Learning Networks

Given an empirical distribution *q* corresponding to a set of data *X*, it is a classical goal to determine a network with *X* as memories. Important for applications is that the network has the ability to denoise a corrupted version of x∈X by converging dynamics; that is, the network functions as an error-correcting coding scheme. Moreover, a practical desire is to estimate such networks from noisy data.

Various scalable approaches to solving this problem are briefly summarized in Table 1. We shall compare these all on the task of learning cliques in Figure 3.

To provide motivation for MEF, we explain its connection to density estimation. Given a data distribution $q={\left(q_{x}\right)}_{x\in {\left\{0,1\right\}}^{n}}\in R^{2^{n}}$, it is natural to try and minimize ∥q−p∥, where *p* is the Lenz–Ising model (2) parameterized by (W,θ), and ∥·∥ is a norm between vectors in $R^{2^{n}}$. It is not clear that accomplishing this would determine networks that have *X* as attractors, but as we will see, it can be useful for such purposes. One difficulty in dealing with such a minimization is that the state space ${\left\{0,1\right\}}^{n}$ is exponential in the number of nodes *n*; in particular, even if the support of *q* is small (i.e., few nonzero coordinates), an exponentially large partition function *Z* is involved.

A subtle modification of the above optimization is the idea to minimize the difference between data and its projection onto the model distribution:

(6) $$\min_{W,\theta }∥q-\frac{\langle q,p\rangle }{\langle p,p\rangle }p∥.$$

Although still intractable, we shall see that the quantity to be minimized in (6) is bounded above by the energy flow EF (Definition 1), which is significantly easier to optimize.

### 3.4. Minimum Energy Flow

Given a binary pattern *x*, let $N_{1}\left(x\right)$ be the set of all those binary vectors one bit different from *x*. We learn Hopfield networks from data having empirical distribution *q* by minimizing the following objective function [21].

**Definition** **1.** *(Energy Flow). The energy flow EF is:*

(7) $$EF\left(W,\theta \right)=\sum_{x\in X}q_{x}\sum_{x^{\prime }\in N_{1}\left(x\right)}e^{(E_{x}-E_{x^{\prime }})/2}.$$

There are several ways to motivate minimizing energy flow (7) to fit networks. Aside from several experimental [21,30,31,32,33,34,35] and theoretical [19] works detailing its utility and properties, a direct explanation is that provably making EF small forces *X* to be attractors of the network (if they can be). It should be somewhat surprising that minimizing (7) forces nonlinear identities (4) of the dynamics.

We present a mathematical derivation of energy flow EF, making its genesis somewhat less ad hoc. Instead of working directly with the projection objective (6), we shall dominate it with the energy flow (7).

**Theorem** **1.** *The energy flow objective EF satisfies the inequality:*

(8) $${∥q-\frac{\langle q,p\rangle }{\langle p,p\rangle }p∥}_{2}\leq \frac{2}{\sigma_{2}}EF,$$

*in which*$\sigma_{2}$*is the second smallest singular value of a certain matrix M (defined in Section 5)*.

The relation above is rather striking; proximity of data *q* to its projection onto the full Lenz–Ising model (2) is bounded by a multiplication of a (data-sized) positive sum of exponential-linear functions with a single structural statistic $\sigma_{2}^{-1}>0$.

We shall prove Theorem 1 in Section 5 using a useful matrix inequality of independent interest (Proposition 1).

### 3.5. Properties

We outline various properties of estimating neural networks from data using MEF. First, note that as EF (7) is a positive sum of exponential-linear functions, it is convex in its parameters [51]. Additionally, EF has a number of terms that are bilinear in the node count and size of data. The networks found by minimizing energy flow determine probability distributions via (2) and the inequality (8) gives a relationship between the objective and distance from data to model. This allows for the estimation of large Lenz–Ising models that model the experimental data well [34,35]; see also Section 4.1.

Minimizing the energy flow determines robust networks that can uncover clean sources from noisy data (see Figure 1, Figure 2 and Figure 3). In special cases, one can even minimize the objective function exactly to analytically answer unsolved classical problems such as proving robust exponential storage in Hopfield networks [19] (Theorem 2). MEF also finds near-optimal solutions to rate-distortion problems for natural signals [30,32,33]. Moreover, MEF exhibits improved learning and generalization versus classical rules as is shown in Figure 3 (see also [21]).

Finally, MEF is a *local rule* in that a weight changes (resp. threshold) only as a function of feedforward input to its two connected nodes. This last property deserves further discussion.

### 3.6. Minimizing Energy Flow Is Biologically Plausible

We call a descent down the gradient of the energy flow (7) given a single pattern X={x} the *MEF learning rule*. Weight and threshold changes for one step are:

(9) $$\begin{array}{cc} \Delta W_{ij} & \propto -x_{j}\Delta x_{i}\exp\left(\Delta x_{i}F_{i}/2\right)=-x_{j}\Delta x_{i}\exp\left(-\Delta E_{i}/2\right),\\ \Delta \theta_{i} & \propto \Delta x_{i}\exp\left(\Delta x_{i}F_{i}/2\right)=\Delta x_{i}\exp\left(-\Delta E_{i}/2\right). \end{array}$$

Here, $F_{i}=W_{i}^{⊤}x-\theta_{i}$ is the *feedforward input* to node *i*. Note that weight changes above are not symmetric. Since the energy function is linked to attractor dynamics, it is only important that we have the same energy function but with symmetric weights. Thus, weight changes are symmetrized to achieve this: $(\Delta W+\Delta W^{⊤})/2$. As these directions descend the gradient of a smooth convex function, traversing them can be very fast [52].

Rule (9) is local and can be understood as a combination of plasticity mechanisms found in biological neural networks. Four cases can be distinguished, depending on the activity of nodes *i* and *j*. When neurons are opposite, it can be interpreted as an induction of *long-term depression* (LTD) mediated by presynaptic activity in the absence of postsynaptic activity. On the other hand, when both are active, the effect is *long-term potentiation* (LTP) mediated by coincident pre- and postsynaptic activity. The negative exponent in the weight update here can be interpreted as a form of *homeostatic plasticity* (HSP): the stronger the postsynaptic cell is activated (measured by the feedforward input $F_{i}$), the stronger the effect of synaptic potentiation is attenuated [53].

### 3.7. Extensions

There are a number of ways to modify the preceding. Larger Hamming neighborhoods $N_{h}$ can be incorporated (e.g., double bit flip neighborhoods $N_{2}$) as well as adding regularizers to the objective function such as an $\ell_{1}$-norm constraint. Moreover, other discrete dynamical systems can be incorporated into this framework (e.g., Potts models [54]). We also note that the energy flow objective can be extended so that higher-order correlations (e.g., third-order Lenz–Ising models) can be captured by MEF.

Inspiration for minimizing energy flow [21] as an objective to learn Hopfield networks is the density estimation work of [55]. Although the MEF objective function presented here and that of [55] are similar, the latter has the property that it is identically zero for data with full support (i.e., all binary vectors appear in the data).

## 4. Results

**Figure 4:**
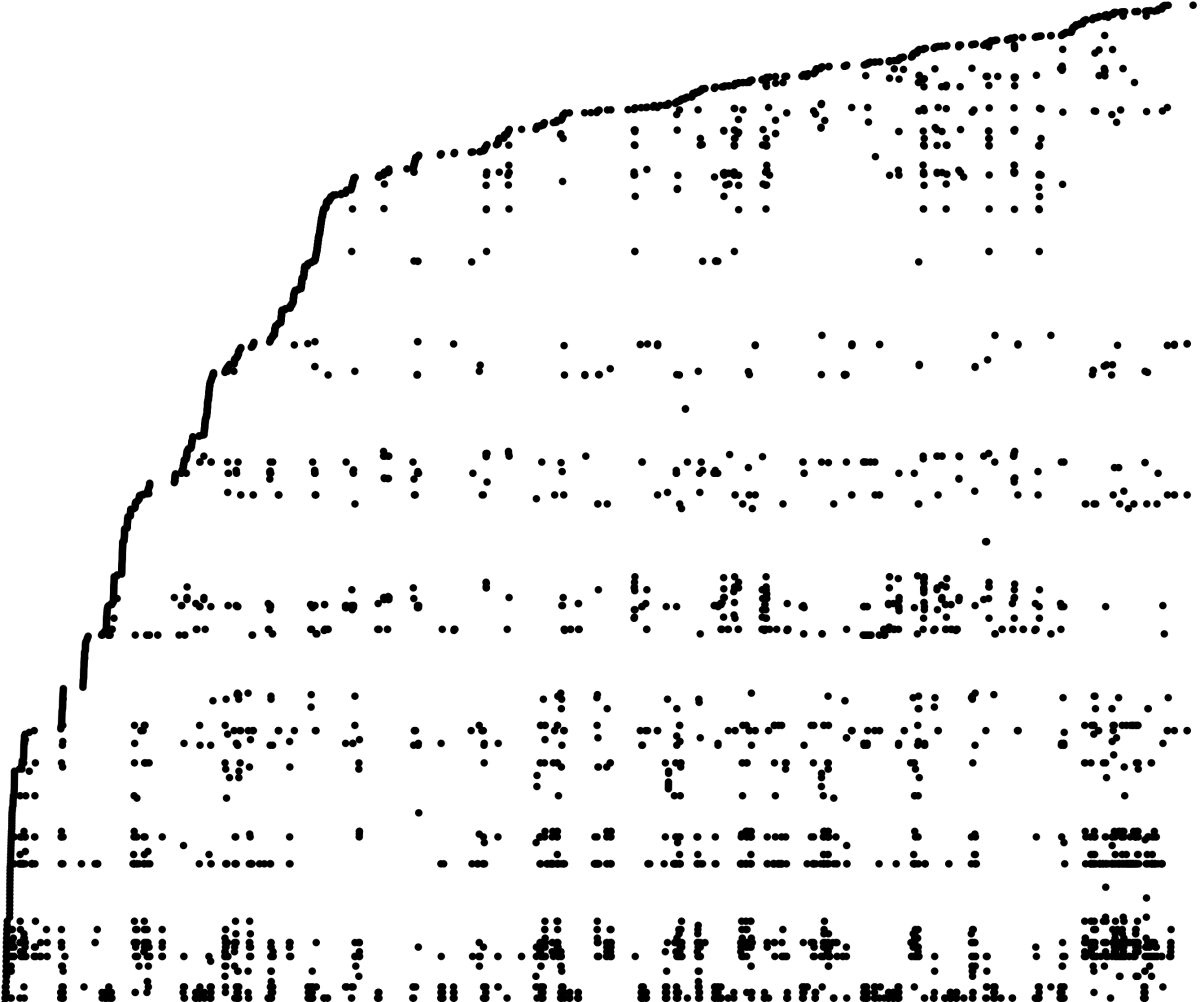
Hidden neural activity. A polytrode recording [56] is analyzed using Hopfield networks. Binary windows of size 50×50, corresponding to 50 neurons and 50 consecutive 2 ms time bins, are extracted from spike timings to train 2500-bit networks with MEF. Over 90 s of data, each circle in the figure represents an attractor initialized at the 100 ms long 50×50 spatiotemporal window of activity starting at a bin. The vertical height of a circle is the logarithm of the corresponding attractor’s first appearance in sequential order; the horizontal position indicates the time bin (left-to-right). Repeating circles along a horizontal line suggest reoccurring neural activity.

**Figure 5:**
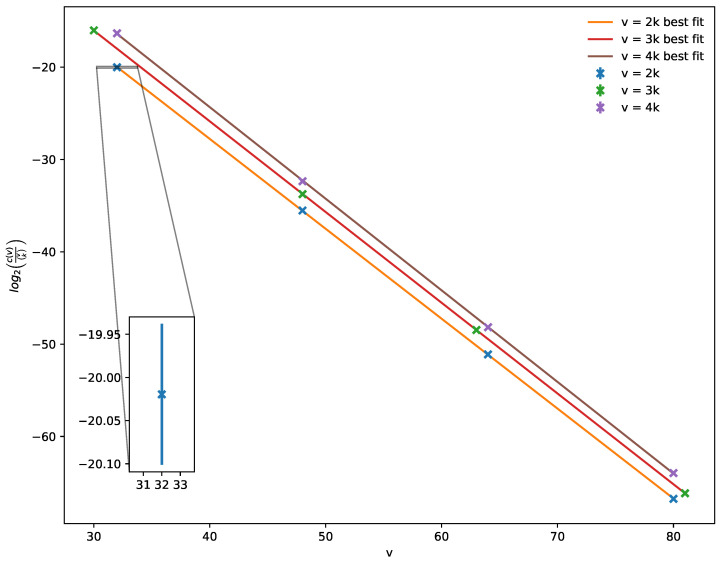
Critical learning of cliques in Hopfield networks. For each value of v=32,48,64,80 (and different v/k=2,3,4) over five trials, the logarithm of the ratio of the number of training *k*-cliques vs. all to achieve a critical 50% accuracy (see [19], Figure 2) on 1000 test cliques is plotted.

Our main results are the following. We use MEF to train a Hopfield network over a full recording of spontaneous spike data and reveal reoccuring spatiotemporal activity patterns in the neural activity (Figure 4). We construct Hopfield networks with robust exponential memory in hypergraphs, and we show that MEF can be used to efficiently learn them (Theorem 2). These networks also naturally define new error-correcting codes (Corollary 1). In the case of graphs, there is a critical ratio of samples when the networks generalize, and we find that it decays exponentially in the number of vertices (Figure 5). We used the Python package [36] to train networks with MEF and perform analyses.

### 4.1. Experimental Neuroscience

We extend the work of [34] and learn a network over all 5 min of data and all neurons in a polytrode recording [56] through layers from an anesthetized cat visual cortex area 18. The result of the analysis is presented in Figure 4 and suggests significant repetition of neural activity in the spike train, uncovered by tracking the sequence of fixed-point (memory) labels as they appear in the data over time (each black circle represents a single 100 ms spatiotemporal window of activity). Note that the method is deterministic and thus gives canonical features for a data set as well as Lenz–Ising parameter estimates. See also [57] for another modern approach to finding structure in neural data.

### 4.2. Hypergraph Codes

We generalize clique learning [19] to the case of hypergraphs. Recall that robust storage is the ability to recover each *n*-bit memory almost surely as n→∞, given a probability *p* that there is an error at each node, whenever *p* is less than some positive best constant α>0, called the index of robustness (see Section 3.2).

The theory from [19] shows that it is possible to store $2^{\Omega (\sqrt{n})}$ memories with robustness index α=1/2. We will prove that for every *d*, there is a Hopfield network that stores $2^{\Omega \left(n^{d/(d+1)}\right)}$ memories robustly. When d=1, this recovers the result of [19].

**Theorem** **2.** *(A) For every* d≥1 *, there exists a Hopfield network on n nodes that stores* $2^{\Omega (n^{c})}$ *memories robustly, where* c=d/(d+1) *. The index of robustness satisfies:*

(10) $$\alpha =\frac{1}{2^{d}\left(d+1\right)-2d}.$$

*(B) Such a Hopfield network can be trained using the MEF rule with index of robustness:*

(11) $$\alpha =\frac{1}{2^{d+1}\left(d+1\right)-4d}.$$

The following is a direct application to the theory of error-correcting codes.

**Corollary** **1.** *For any ϵ>0, there exist n-node Hopfield networks that error-correct $2^{\Omega (n^{1-\epsilon })}$ patterns through a binary symmetric channel with crossover probability α given by (10).*

As the proof will show, Theorem 2 is true even with only a single synchronous iteration of the dynamics. In particular, memories corrupted with αn bits of error on average can be corrected from a single parallel recurrent pass through all nodes.

### 4.3. Critical Learning

The following computational result illustrated in Figure 5 demonstrates critical learning in Hopfield neural networks. In [19] (Figure 2), it was experimentally shown that there is a critical number of training samples at which Hopfield networks trained with MEF on random subsets of *k*-cliques in graphs on v=2k vertices store all such cliques. We extend this finding by computing for large graphs the ratio of this critical number c(v) of samples to total number vk of *k*-cliques; the result is that the ratio decays exponentially in the number of vertices.

**Conjecture** **1.** *The critical ratio c(v)/vk for learning all k-cliques in graphs on v vertices using MEF decays exponentially in the number of vertices v=2k.*

Theoretical verification of this conjecture is the focus of future work.

## 5. Proofs

We provide complete proofs of the mathematical results stated in Theorems 1 and 2.

### 5.1. MEF Inequality

Before proving inequality (8) from Theorem 1, we first need to state a basic fact relating projections onto principal eigenvectors of a positive semidefinite matrix.

**Proposition** **1.** *Let $A\in R^{n\times n}$ be an n×n singular positive semidefinite matrix and let $\left\{u_{1},\ldots ,u_{n}\right\}$ be an orthonormal set of eigenvectors of A corresponding to eigenvalues $0=\lambda_{1}\leq \ldots \leq \lambda_{n}$. Suppose that the rank of A is n−1 (so that $\lambda_{2}>0$). Then, for any $x\in R^{n}$, we have:*

(12) $$∥x-\langle x,u_{1}\rangle u_{1}{∥}_{2}^{2}\leq \frac{x^{⊤}Ax}{\lambda_{2}}.$$

**Proof.**  Since $\left\{u_{1},\ldots ,u_{n}\right\}$ is an orthonormal basis of $R^{n}$, we can write $x=\sum_{i=1}^{n}\alpha_{i}u_{i}$, for real numbers $\alpha_{i}=\langle x,u_{i}\rangle$. A straightforward computation gives:

(13) $$x^{⊤}Ax=\sum_{i=2}^{n}\alpha_{i}^{2}\lambda_{i}\geq \lambda_{2}||x-\langle x,u_{1}\rangle u_{1}{\left|\right|}_{2}^{2}.$$

Rearranging produces the inequality in the theorem statement.    □

**Corollary** **2.** *Suppose that a $2^{n}\times 2^{n}$ matrix M has eigenvector p and second smallest singular value $\sigma_{2}>0$. Then,*

(14) $${∥q-\frac{\langle q,p\rangle }{\langle p,p\rangle }p∥}_{2}\leq \frac{{∥Mq∥}_{2}}{\sigma_{2}}\leq \frac{{∥Mq∥}_{1}}{\sigma_{2}}.$$

**Proof.**  Set $u_{1}=\frac{p}{{\langle p,p\rangle }^{1/2}}$ with $A=M^{⊤}M$ in Proposition 1, take the square root of both sides, and use the inequality ${∥\cdot ∥}_{2}\leq {∥\cdot ∥}_{1}$.    □

**Proof of Theorem** **1.** There are several ways to construct a matrix *M* so that we can apply Corollary 2. One move is to define (recall $N_{1}$ from Section 3.4):

(15) $$M_{yx}=e^{(E_{x}-E_{y})/2},y\in N_{1}\left(x\right);M_{yx}=0,x\neq y\notin N_{1}\left(x\right);M_{xx}=-\sum_{y\neq x}M_{yx}.$$

This matrix has column sums zero, and it can be readily checked that *p* as in (2) is an eigenvector with eigenvalue 0 for *M* since it satisfies *detailed balance*:

(16) $$M_{xy}p_{y}=M_{yx}p_{x}.$$

Note also that the graph for the matrix *M* is connected (so that $\sigma_{2}\left(M\right)>0$).

Let us examine the right-hand side of inequality (14) in light of this choice of *M*. Decompose M=D+T into a nonpositive diagonal matrix *D* and a nonnegative matrix *T* with zeroes on its diagonal. From the triangle inequality, we have:

(17) $${∥Mq∥}_{1}\leq {∥Dq∥}_{1}+{∥Tq∥}_{1}.$$

Note that *T* and *q* are both nonnegative so that (1 is the all ones vector):

(18) $${∥Tq∥}_{1}=\langle 1,Tq\rangle =\langle T^{⊤}1,q\rangle ={∥Dq∥}_{1}={∥\left(-D\right)q∥}_{1}=\sum_{x}q_{x}\sum_{x^{\prime }\in N_{1}\left(x\right)}M_{x^{\prime }x}=EF.$$

The inequality (8) now follows directly from combining (14), (17), and (18).    □

### 5.2. Hyperclique Theorem

Our approach is inspired by [19], which proceeded by defining nodes of a Hopfield network to correspond to possible edges on a vertex set, with memories corresponding to certain graphs on that vertex set. In our case, nodes will correspond to hypercliques on a vertex set, and memories will correspond to hypergraphs on that vertex set.

Consider a set *V* of *v* vertices and define a corresponding Hopfield network on n=vd+1 nodes, where each node *i* corresponds to a (d+1)-element subset $V_{i}\subset V$ (the case of d=1 is analogous to the approach of [19]). Note that for clarity throughout, we will use *nodes* to refer to neurons of the Hopfield network, and *vertices* to refer to the elements of the underlying set *V* used to define the network.

Given a node *i* and a *d*-uniform hypergraph *G* on vertex set *V*, we say that *i* is *complete* (otherwise *incomplete*) if the corresponding subset $V_{i}$ of *V* is a *hyperclique*; that is, if all *d*-element subsets of $V_{i}$ are hyperedges of *G*. We define x(G) to be the assignment x of states such that $x_{i}=1$ if and only if *i* is complete.

Our goal will be to set weights such that the set of memories contains x(G) for almost all *d*-uniform hypergraphs *G* on the vertex set *V*, and that these memories are stored robustly. Since the number of *d*-uniform hypergraphs on vertex set *V* is $2^{\Theta (v^{d})}$, the number of memories of the Hopfield network will be $2^{\Omega (v^{d})}$. Because n=vd+1=Θ(vd+1), we have $2^{\Omega (v^{d})}=2^{\Omega (n^{d/(d+1)})}$, as desired.

For nodes i,j in the Hopfield network, we write i∼j if we have $|V_{i}\cap V_{j}|=1$. We write w(G,i) for the number j∼i such that *j* is complete. We will consider the set *S* of graphs *G* such that w(G,i) satisfies the following conditions:

1. If *i* is complete,
  (19) $$w\left(G,i\right)=\frac{(d+1)v}{2^{d}}\left(1\pm o\left(1\right)\right).$$
2. If *i* is incomplete,
  (20) $$w\left(G,i\right)=\frac{dv}{2^{d}}\left(1\pm o\left(1\right)\right).$$
3. The number of complete *i* is
  (21) (1±o(1))2−(d+1)vd+1.

**Lemma** **1.** *With probability 1−o(1), a random d-uniform hypergraph G is in S.*

**Proof.**  First, we consider the probability that condition 1 holds. Let us suppose that a certain (d+1)-hyperclique *i* is present in *G*, but no other hyperedges are known. Consider a hyperedge-exposure martingale $X_{k}$, where the remaining hyperedges of *G* are presented in some order, and $X_{k}$ represents the expected value of w(G,i) after revealing which of the first *k* hyperedges are present.

Note that $X_{k}\neq X_{k-1}$ if and only if the hyperedge last revealed is present in some hyperclique j∼i. This hyperedge must share d−1 vertices with *i*, which means that it is an element of exactly two such hypercliques *j*. Therefore, $|X_{k}-X_{k-1}|\leq 2$. Applying the Azuma–Hoeffding inequality [58,59], we can upper bound the probability that w(G,i) deviates markedly from expectation:

(22) $$\mathrm{Pr}\left[\left|w\left(G,i\right)-\frac{(d+1)v}{2^{d}}\right|>\sqrt{v}\log v\right]\leq \exp\left[{\left(\log v\right)}^{2}/8\right].$$

Thus, the probability this condition holds for every *i* is at most vd+1exp[(logv)2/8].

We now consider the probability that condition 2 holds. As before, suppose that a certain (d+1)-hyperclique *i* is present in *G* and that we know its hyperedges but no others. Consider a hyperedge-exposure martingale $X_{k}$, where the remaining hyperedges of *G* are presented in some order and $X_{k}$ represents the expected value of w(G,i) after revealing which of the first *k* hyperedges are present. Once more, $|X_{k}-X_{k-1}|\leq 2$, and we obtain the same bound on the probability of condition 2 as in condition 1.

Finally, we consider the probability that condition 3 holds. Consider a hyperedge-exposure martingale $X_{k}$ where all the hyperedges of *G* are presented in some order and $X_{k}$ represents the expected number of (d+1)-hypercliques after revealing which of the first *k* hyperedges are present. Note that $|X_{k}-X_{k-1}|<v$, since at most *v* hypercliques can contain a certain hyperedge. Now we apply the Azuma–Hoeffding inequality:

(23) Pr#of hypercliques−2−(d+1)vd+1>v3/2logv≤exp[(logv)2/2].

Combining our results together, we find that the probability that none of conditions 1, 2, and 3 are violated is at most:

(24) vd+1exp[(logv)2/8]+vd+1exp[(logv)2/8]+exp[(logv)2/2]=o(1).

   □

It thus suffices to prove that every element of *S* is stored robustly by our Hopfield network. To simplify the model, we will suppose that all weights are a constant x≥0 for i∼j and otherwise 0. We will also assume that $\theta_{i}$ equals *z* for every *i*. See [19] (Section 5.1) for more detail on such symmetry considerations.

Consider G∈S, and let *i* be a node of the network.

**Proof of Theorem** **2.** In order to prove robust storage, we must consider two sets of conditions. First, fixed-point conditions are needed to ensure that every element of *S* is indeed a memory. There are two cases to be considered.

If *i* is complete, then we require $x_{i}=1$ to be preserved by the dynamics. This condition is equivalent to w(G,i)x−vz>0. From the definition of *S*, we have $w\left(G,i\right)\geq \frac{(d+1)v}{2^{d}}\left(1-o\left(1\right)\right)$, so it suffices to satisfy:

(25) $$0<\frac{(d+1)v}{2^{d}}\left(1-o\left(1\right)\right)\cdot x-vz.$$

Alternatively, if *i* is incomplete, then we require $x_{i}=0$ to be preserved by the dynamics, given by $w_{1}x-vz<0$. From the definition of *S*, we have $w\left(G,i\right)\leq \frac{dv}{2^{d}}\left(1+o\left(1\right)\right)$, so it suffices to satisfy:

(26) $$0>\frac{dv}{2^{d}}\left(1+o\left(1\right)\right)\cdot x-vz.$$

Next, we shall need conditions to ensure that every α-corrupted element of *S* is reconstructed under the dynamics. We will work with the stronger condition that the reconstruction takes place in a single step. Let $w^{\prime }\left(G,i\right)$ denote the number of j∼i such that *j* is incomplete; thus, $w\left(G,i\right)+w^{\prime }\left(G,i\right)=\left(d+1\right)\left(v-\left(d+1\right)\right)$. Then, after corruption, the number of j∼i such that $x_{j}=1$ is given by:

(27) $$\begin{array}{cc} p\cdot w^{\prime }\left(G,i\right)+\left(1-p\right)\cdot w\left(G,i\right) & =\left(1-2p\right)\cdot w\left(G,i\right)+p(w\left(G,i\right)+w^{\prime }\left(G,i\right))\\ \; & =(1-2p)\cdot w(G,i)+p(d+1)(v-(d+1)). \end{array}$$

Once again, there are two cases.

If *i* is complete, then we require $x_{i}=1$ to be recovered by the dynamics, so we must have:

(28) $$0<\left(\left(1-2p\right)\cdot w_{1}+p\left(d+1\right)\left(v-\left(d+1\right)\right)\right)x-vz.$$

It thus suffices to satisfy:

(29) $$\begin{array}{cc} 0 & <\left(\left(1-2p\right)\cdot \frac{(d+1)v}{2^{d}}\left(1-o\left(1\right)\right)+p\left(d+1\right)\left(v-\left(d+1\right)\right)\right)x-vz\\ \; & =\left(\left(1-2p\right)\cdot \frac{dv}{2^{d}}+p\left(d+1\right)v\right)\left(1-o\left(1\right)\right)x-vz. \end{array}$$

This inequality follows immediately from (25), since we have assumed x≥0 and d≥1.

Alternatively, if *i* is incomplete, then we require $x_{i}=0$ to be recovered by the dynamics, so we must have:

(30) $$0>\left((1-2p)\cdot w(G,i)+p(d+1)(v-(d+1))\right)x-vz.$$

It thus suffices to satisfy:

$$\begin{array}{cc} 0 & >\left(\left(1-2p\right)\cdot \frac{dv}{2^{d}}\left(1+o\left(1\right)\right)+p\left(d+1\right)\left(v-\left(d+1\right)\right)\right)x-vz\\ \; & =\left(\left(1-2p\right)\cdot \frac{dv}{2^{d}}+p\left(d+1\right)v\right)\left(1+o\left(1\right)\right)x-vz. \end{array}$$

This inequality immediately implies (26), where we again use x≥0 and d≥1.

We conclude that robust storage is satisfied if and only if both (25) and (31) are satisfied. Since we can pick *x* and *z* arbitrarily, it suffices to have:

(31) $$\frac{(d+1)v}{2^{d}}\left(1-o\left(1\right)\right)>\left(\left(1-2p\right)\cdot \frac{dv}{2^{d}}+p\left(d+1\right)v\right)\left(1+o\left(1\right)\right).$$

This inequality reduces to

(32) $$p<\frac{1-o(1)}{2^{d}\left(d+1\right)-2d},$$

proving part (1) of Theorem 2.

In order to prove part (2), we must find the minimum of the expression for energy flow:

(33) $$\frac{1}{\left|S\right|}\sum_{x(G)|G\in S}\sum_{x(G,i)|i\in V}\exp\left[\frac{E_{x(G)}-E_{x(G,i)}}{2}\right],$$

where x(G,i) denotes the state of the Hopfield network in which $x_{i}$ is switched from the state x(G). For a given value of *z*, we wish to find the value of *x* such that (33) is minimized. For each choice of (G,i) in the summand, there are two possibilities. If *i* is complete, then $E_{x(G)}-E_{x(G,i)}=-w\left(G,i\right)\cdot x+z$. If *i* is incomplete, then $E_{x(G)}-E_{x(G,i)}=w\left(G,i\right)\cdot x-z$.

Thus, we seek to minimize the following with respect to *x*:

(34) $$\frac{1}{\left|S\right|}\sum_{G\in S,\;i\;\mathrm{complete}}\exp\left[\frac{-w(G,i)\cdot x+z}{2}\right]+\frac{1}{\left|S\right|}\sum_{G\in S,\;i\;\mathrm{incomplete}}\exp\left[\frac{w(G,i)\cdot x-z}{2}\right].$$

Leaving off the initial constant and taking the derivative with respect to *x*, we seek *x* satisfying:

(35) $$\begin{array}{cc} 0= & \sum_{G\in S,\;i\;\mathrm{complete}}-w\left(G,i\right)\exp\left[\frac{-w(G,i)\cdot x+z}{2}\right]\\ \; & \;+\sum_{G\in S,\;i\;\mathrm{incomplete}}w\left(G,i\right)\exp\left[\frac{w(G,i)\cdot x-z}{2}\right]. \end{array}$$

It is simple to verify that this critical point for *x* exists uniquely and represents a global minimum.

From the definition of *S*, we have:

(36) $$\begin{array}{cc} 0= & \sum_{G\in S,\;i\;\mathrm{complete}}-w\left(G,i\right)\exp\left[\frac{-w(G,i)\cdot x+z}{2}\right]\\ \; & +\sum_{G\in S,\;i\;\mathrm{incomplete}}w\left(G,i\right)\exp\left[\frac{w(G,i)\cdot x-z}{2}\right]\\ = & \sum_{G\in S,\;i\;\mathrm{complete}}-\frac{(d+1)v}{2^{d}}\left(1\pm o\left(1\right)\right)\exp\left[\frac{-\frac{(d+1)v}{2^{d}}\left(1\pm o\left(1\right)\right)\cdot x+z}{2}\right]\\ \; & +\sum_{G\in S,\;i\;\mathrm{incomplete}}\frac{dv}{2^{d}}\left(1\pm o\left(1\right)\right)\exp\left[\frac{\frac{dv}{2^{d}}\left(1\pm o\left(1\right)\right)\cdot x-z}{2}\right]. \end{array}$$

Again from the definition of *S*, we know the approximate number of complete *i* for each *G*, giving us:

(37) (1±o(1))2−(d+1)vd+1·(d+1)·exp−(d+1)v2d(1±o(1))·x+z2=(1±o(1))1−2−(d+1)vd+1·d·expdv2d(1±o(1))·x−z2.

Simplifying, we obtain:

(38) $$\exp\left[\frac{-\frac{(2d+1)v}{2^{d}}\left(1\pm o\left(1\right)\right)\cdot x}{2}+z\right]=\left(1\pm o\left(1\right)\right)\left(2^{d+1}-1\right)v\cdot \frac{d}{d+1}.$$

Thus, we have:

(39) $$x=\left(1\pm o\left(1\right)\right)\frac{2^{d+1}}{(2d+1)v}\cdot \left(z-(1\pm o(1))(d+1)\ln2\right).$$

We obtain the same expression if we minimize energy flow with respect to *z* while holding *x* constant. Therefore, the minimum occurs for any *x* and *z* satisfying the above equation. By picking a *z* that is large enough, we find that a minimum occurs at:

(40) $$x=\left(1\pm o\left(1\right)\right)\frac{2^{d+1}}{(2d+1)v}z.$$

This setting for *x* and *z* satisfies (25) and (31) if we have:

(41) $$p<\left(1-o\left(1\right)\right)\frac{1}{2^{d+1}\left(d+1\right)-4d},$$

completing our proof of Theorem 2.    □

Note that in the case d=2, the theorem shows that a Hopfield network can reconstruct almost every graph from its set of triangles, even with significant corruption.

## 6. Discussion

Although we are motivated by problems involving memory storage and capacity for recurrent networks, there are several other applications (Section 2) of the methods and results (Section 4) presented here. For example, unsupervised clustering and denoising can be used to understand experimental data coming from science (Figure 4), and new DRNN-based error-correcting codes are poised for practical effect (Corollary 1).

The findings here also suggest hypotheses of synaptic adaptation in neuroscience that can be verified experimentally. In particular, it is possible to dissociate between the different learning rules found in Table 1. One intriguing possibility arising from this work is that minimizing energy flow is a scalable approximation to the powerful (but intractable) maximum likelihood estimation for adjusting synaptic strength in neurons.

There are several directions to take this work further. For instance, it would be interesting to generalize to other combinatorial patterns sets, as well as incorporate the full McCulloch–Pitts time-series model [60]. It is also possible to view MEF learning of robust pattern storage in the context of Probably Approximately Correct (PAC) theory [61] from computer science, but we have not explored the connection fully.

Finally, the concept of criticality has deep ties to neuroscience and complex systems theory [62,63] and is believed to be an important signature of intelligent systems performing a computation. With Figure 5 and Conjecture 1, we suggest that critical learning might be a key property of Hopfield networks. In particular, full generalization of the networks to unseen patterns appears to take place at sharp phase transitions.

## 7. Conclusions

Minimizing energy flow to learn parameters in Hopfield networks has applications in memory capacity, unsupervised clustering, signal modeling, error-correcting codes, graph theory, and neuroscience. Moreover, networks determined using the convex MEF objective are dissociable from classically trained ones and display characteristics such as locality, homeostasis, scalability, robustness, and generalization.

## Figures and Tables

**Table 1:** Learning rules to train Hopfield networks of binary linear threshold neurons.

Learning Rule	Principle
Outer-product (OPR)	Hebb’s rule sets weights to be correlation
Perceptron	Supervised pattern memorization
Delta	Least mean square objective function
Contrastive divergence	Maximum likelihood estimation by sampling
Minimum energy flow (MEF)	Approximate maximum likelihood estimation

## Data Availability

Data from experimental neuroscience analyzed in this work can be found at CRCNS https://crcns.org.

## Abbreviations

   The following abbreviations are used in this manuscript:

MEF	minimum energy flow
MLE	maximum likelihood estimation
OPR	outer product rule
DRNN	discrete recurrent neural network
